# Supplementary material for: NEBULA101: an open dataset for the study of language aptitude in behaviour, brain structure and function
Source: Sci Data. 2025 Jan 6;12:19. doi: 10.1038/s41597-024-04357-y (PMC11704325; doi:10.1038/s41597-024-04357-y)
Supplement: Supplementary file 1 — Supplementary Information File [file 41597_2024_4357_MOESM1_ESM.pdf]

Supplementary information v2.0 OCT-24: NEBULA101: an open dataset for the study of language aptitude in behaviour, brain structure and function

A. Rampinini, I. Balboni, O. Kepinska, R. Berthele, N. Golestani

Supplementary Information File

Supplementary information v2.0 OCT-24: NEBULA101: an open dataset for the study of language aptitude in behaviour, brain structure and function

A. Rampinini, I. Balboni, O. Kepinska, R. Berthele, N. Golestani

This file contains supplementary information for the paper **NEBULA101: an open dataset for the study of language aptitude in behaviour, brain structure and function**

For details, please refer to the main article.

Figure S1. Output of BIDS validation (screen-print from the LINUX Terminal):

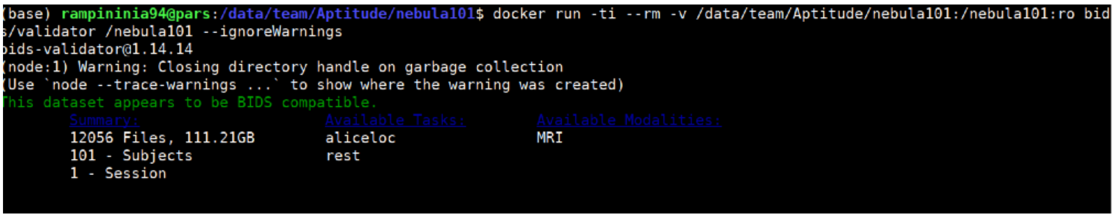

Supplementary information v2.0 OCT-24: NEBULA101: an open dataset for the study of language aptitude in behaviour, brain structure and function

A. Rampinini, I. Balboni, O. Kepinska, R. Berthele, N. Golestani

Table S1: overview of all tests and modalities, with details specific to this dataset. For the complete bibliography, please refer to the main article, Table 2.

Test	*Modality*	*Construct*	*Delivery*	*Instructions*	*Derived score*	*Specifications*
Language Experience and Proficiency Questionnaire (LEAP-Q)	Questionnaire	Multilingual language experience.	Online, unsupervised	Fill in the provided form.	Entropy	Extended to accommodate up to 50 languages.<br>Added questions on:<br>*Time spent* in contexts such as online communities, fandoms and subcultures where a language is spoken.<br>*Contributors* to language learning: social media, apps and everyday life in the country where a language is spoken.<br>Removed question on cultural identification.<br>French adaptation.
Code Switching questionnaire	Questionnaire	Code switching habits.	Online, unsupervised	Fill in the provided form.	Voluntary switching<br>Involuntary switching	Selected only voluntary and involuntary switching scores.<br>French adaptation.
Motivational Factors Questionnaire (MFQ)	Questionnaire	Motivation and attitude towards foreign languages (FL).	Online, unsupervised	Fill in the provided form.	Ideal foreign language self<br>Instrumentality<br>International contact<br>Foreign language interest<br>Foreign language anxiety<br>Foreign language confidence<br>Milieu<br>Usage willingness of foreign languages	French adaptation.<br>5-point Likert scale instead of 6-point.<br>Constructs/items assuming that participants are actively involved in studying foreign languages (academically) were not included.<br>Items that refer specifically to the English language, anglophone world and culture reworded to fit a more global context where possible or excluded.<br>Included constructs are shown in the Derived Score column.
Adult Reading History Questionnaire (AHRQ)	Questionnaire	Reading history.	Online, unsupervised	Fill in the provided form.	Reading history score	French version of the questionnaire.

Internal Representations Questionnaire (IRQ)	Questionnaire	Modes of internal reasoning.	Online, unsupervised	Fill in the provided form.	Manipulation Factor<br>Orthographic Factor<br>Verbal Factor<br>Visual Factor	French adaptation.
Music Use and Background Questionnaire (MUSEBAQ)	Questionnaire	Music training, capacity, preferences, and motivations.	Online, unsupervised	Fill in the provided form.	Index of music training<br>Index of music listening<br>Index of musical instrument playing<br>Cognitive and Emotional Regulation<br>Social Connection<br>Engaged Production<br>Dance<br>Physical Exercise	French adaptation.
Barratt's Simplified Measure of Socioeconomic Status (BSMSS)	Questionnaire	Socioeconomic status.	Online, unsupervised	Fill in the provided form.	Barratt's Simplified Measure of Socioeconomic Status	French adaptation.
Edinburgh Handedness Inventory (EHI)	Questionnaire	Handedness.	Online, unsupervised	Fill in the provided form.	Handedness score	10-item version.
Artgram	Behavioural task	Language analytic abilities / Morphosyntax.	Online, supervised	Study the provided dictionary and choose the appropriate adaptation for given sentences by recognising the use of morphological cues.	Accuracy<br>RT	7-word dictionary to study for 3 minutes.<br>12 sentences to translate, with 4 possible choices, only 1 correct choice.<br>Self-advancement.<br>15-minute time limit.
Modern Language Aptitude Test 5 (MLAT5)	Behavioural task	Rote learning.	Online, supervised	Study the provided dictionary and choose the appropriate adaptation for given words.	Accuracy<br>RT	French adaptation.
Farsi uvular Production Task	Behavioural task	Foreign sound production.	Online, supervised	Listen to and reproduce words containing a foreign	Accuracy	n/a

				language sound not present in French.		
Hindi Dental Retroflex Contrast	Behavioural task	Phonological categorisation/discrimination.	Online, supervised	Categorise a foreign language sound not present in French. Training and testing blocks.	Accuracy	200 trials were presented.
Brocanto	Behavioural task	Language analytic abilities / Pattern recognition.	Online, supervised	Recognise grammatical and ungrammatical sentences with different structures and violation types in an artificial language, inductively.<br>Training (reading-only) and testing (judge grammaticality by button press) blocks.	Accuracy<br>RT<br>(For each block 1-3 and overall)	Version from Kepinska et al., 2017 with minor modifications related to timing and condition counterbalancing, as follows:<br>A practice screen was added at the beginning of each testing block to test button press and rule comprehension.<br>Button press instructions: 1 for "grammatical" and 0 for "ungrammatical".<br>We chose a 2-second fixation cross without jittering.<br>We chose a 6-second sentence presentation in both testing and training block types.<br>We maintained 40 sentences per testing block, but we included 8-word sentences and obtained a 1/2 ratio of grammatical and ungrammatical sentences **overall** (N=120, of which the 60 ungrammatical sentences were new and roughly 1/3 of the 60 grammatical ones [N=22] had been presented during training).
Raven's Advanced Progressive Matrices (APM)	Behavioural task	Non-verbal intelligence.	Online, supervised	Select the missing block to complete a picture from a set of proposed choices.	Accuracy<br>RT	Advanced and abridged French version.<br>Time limit of 20 minutes for 23 trials.<br>Programmed for computer-based presentation on the Gorilla platform.
Corsi block	Behavioural task	Visuospatial memory	Online, supervised	Watch a sequence of blocks being highlighted, and repeat the same sequence by clicking on them in the same order and then backwards.	Forward span<br>Backward span<br>Total span	Programmed for computer-based presentation on the Gorilla platform in place of in-person.<br>The blocks were presented as 9 2-dimensional black squares arranged on a white background.<br>Blocks were black.<br>Sample block sequences were shown in

						yellow.<br>Participants clicked on the blocks instead of tapping them with their hand.<br>Selected blocks were shown in green upon clicking.<br>Block placement on the screen followed Kessels et al. 2000[89]
Digit Span	Behavioural task	Auditory working memory	Online, supervised	Recollect the digits immediately after the presentation in the same order, in the 'forward' section of the task, or in the inverse order, in the 'backward' section.	Forward span<br>Backward span<br>Total span	Auditory modality.<br>Response collected through upper numeric keypad.
Revised Tempo Test	Behavioural task	Arithmetic abilities	Online, supervised	Solve 60 additions and 60 subtractions in 1 minute each.	Accuracy<br>RT<br><br>(Additions, subtractions, overall)	Programmed for computer-based presentation on the Gorilla platform in place of paper and pencil, otherwise unchanged.<br>Response collected through upper numeric keypad.
Advanced Measures of Music Audiation (AMMA)	Behavioural task	Music audiation, musicality, musical aptitude.	Online, supervised	Judge difference or identity in melody or rhythm between pairs of musical excerpts.	Tonal score<br>Rhythm score<br>Total score	Programmed for computer-based presentation on the Gorilla platform.
Attention Network Test-Interaction (ANT-I)	Behavioural task	Attention networks: executive control, alerting, orienting.	In person	Detect arrow orientation in presence of flankers and sound cues.	Alerting gain<br>Orienting gain<br>Reorienting gain<br>Inhibition gain	French adaptation (instructions)<br>18 conditions and 432 trials.
California Verbal Learning Task (CVLT)	Behavioural task	Verbal working memory	In person	Remember lists of words short-term and long-term.<br>Recognise previously presented words, long-term.	Accuracy immediate recall<br>Accuracy immediate and short-term recall<br>Accuracy long-term recognition<br>RT long term recognition	Programmed for computer-based presentation on the Gorilla platform.<br>Removed cued recall (short and long-term).

Finger tapping Test	Behavioural task	Fine motor skills	In person	Tap with the index finger of the dominant and then non-dominant hand on the spacebar as fast as possible.	Average finger tapping score dominant hand overall<br>Average finger tapping score nondominant hand overall<br>Average finger tapping score of dominant/nondominant overall<br><br>(Calculated over blocks)	Abbreviated version of the Finger Tapping test as proposed by Ashendorf et al., 2015.<br><br>5 trials per hand.<br>3 dominant hand trials – 1-minute break – 2 dominant hand trials.<br>3 non-dominant hand trials – 1-minute break – 2 non-dominant hand trials.
Purdue Pegboard Test	Behavioural task	Fine motor skills	In person	Insert pegs in holes, first with the dominant and and then with the non-dominant hand.<br><br>Build assembly of pegs alternating hands as instructed.	Accuracy, dominant hand<br>Accuracy, nondominant hand<br>Accuracy, simultaneous hands<br>Accuracy, assembly task<br>Average accuracy, dominant-nondominant-simultaneous hands<br><br>(Calculated over blocks)	French adaptation (instructions)
Rapid Automatised Naming (RAN)	Behavioural task	Naming automatisation	In person	Rapidly denominating objects, digits, colours	Accuracy<br>RT	French adaptation.<br>Programmed for computer-based presentation on the Gorilla platform.
Phoneme suppression	Behavioural task	Phonological awareness	In person	Repeat words by omitting the first phoneme.	Accuracy<br>RT	Programmed for computer-based presentation on the Gorilla platform.<br><br>Pre-recorded female voice.
Text Reading	Behavioural task	Reading skills	In person	Read two texts of increasing difficulty.	Accuracy<br>RT	Programmed for computer-based presentation on the Gorilla platform.
Word and Pseudoword Reading	Behavioural task	Reading skills	In person	Reading lists of words and pseudowords.	Accuracy<br>RT<br><br>(Per stimulus type: regular words, irregular words and pseudowords; overall)	With the target population in mind, this task was made more difficult by merging two standardized dyslexia assessment tests in French, the ECLA16+ and the EVALEC:<br><br>- There were 56 regular words, 52 irregular words, and 56 pseudowords overall.

A. Rampinini, I. Balboni, O. Kepinska, R. Berthele, N. Golestani

						- For each category 20 words were taken from the Ecla16+ and the remaining from the EVALEC.<br>- The words from the EVALEC were randomized within-list rather than mixing across lists, to be able to time and score the lists per word-type (regular, irregular and pseudowords).
Spelling task	Behavioural task	Spelling skills	In person	Write down words, pseudowords and irregular words after hearing them.	Accuracy<br>(regular words, irregular words and pseudowords; overall)	Programmed for computer-based presentation on the Gorilla platform.
Spoonerisms	Behavioural task	Phonological awareness	In person	Swap the first phoneme of word pairs.	Accuracy<br>RT	Programmed for computer-based presentation on the Gorilla platform.<br>Pre-recorded male voice
Non-word repetition	Behavioural task	Phonological working memory	In person	Repeat non-word lists of increasing length.	Accuracy of repetition<br>Span, i.e. maximum number of repeated words	Programmed for computer-based presentation on the Gorilla platform.
Structural MRI (T1-weighted MPRAGE)	sMRI	Brain structural anatomy	In person	Lie still in scanner.	n/a	whole-brain coverage<br>1mm isotropic voxel<br>FOV read = 256mm, FOV phase 93.8%<br>TR = 2300ms, TE = 3.26ms<br>Flip Angle: 9°<br>Distance Factor 50 %<br>Orientation: Sagittal<br>Phase Encoding Direction: A >> P
Diffusion-Weighted Imaging (DWI)	dMRI	Diffusion gradients	In person	Lie still in scanner.	n/a	Multishell sequence:<br>1.5mm isotropic voxel<br>FOV read = 225mm, FOV phase 100%<br>whole-brain coverage<br>TR 6700.0 ms, TE 74.00 ms<br>Distance Factor 0 %<br>Acceleration factor SMS = 2 GRAPPA = 3<br>Orientation: Transversal<br>Phase Encoding Direction: A >> P<br>Diffusion-encoding gradient directions: 117

						12 B_0 volumes distributed along the sequence<br>7 volumes at 700 s/mm2<br>30 volumes at 1000 s/mm2<br>68 volumes at 2850 s/mm2
Language Network Functional Localiser (fMRI)	fMRI	Functional activation for language	In person	Lie still in scanner, eyes open, fixate cross, listen to story	n/a	Added a condition for degraded second language.<br>Added a fixation at the beginning and at the end of each run.
Resting-State Functional MRI (fMRI)	fMRI	Resting-state functional activation	In person	Lie still in scanner, eyes open	n/a	2mm isotropic voxel<br>FOV read = 224mm, FOV phase 100%<br>whole-brain coverage<br>72 slices<br>TR = 2000ms, TE = 32ms<br>Flip Angle: 75°<br>Distance Factor 0 %<br>Acceleration factor SMS = 3<br>Orientation: Transversal<br>Phase Encoding Direction: A >> P
Field map	MRI	Intensity of the magnetic field across space	In person	Lie still in scanner.	n/a	Intended to correct B0 distortion in fMRI:<br>2.4x2.4x2mm^3 voxel<br>FOV read = 225mm, FOV phase 100%<br>whole-brain coverage<br>72 slices (fMRI), 66 slices (DWI)<br>TR = 700ms, TE1 = 4.92ms, TE2=7.38ms<br>Flip Angle: 60°<br>Distance Factor 0 %<br>Orientation: Transversal<br>Phase Encoding Direction: R >> L

A. Rampinini, I. Balboni, O. Kepinska, R. Berthele, N. Golestani

Table S2. Pairwise linear Pearson correlations among variables that reached significance and were above |r(100)| > .5. For brevity, between scores having a 'corr' and 'incorr' (correct ,incorrect) columns, only 'corr' was selected. Age and education were not considered. Correlations across tasks are marked with * and ** for direct and inverse, respectively.

Construct(s)	Test(s)	Variable 1	Variable 2	Correlation ($r_{Pearson}$)
Verbal working memory: immediate and total recall	CVLT	cvlt_tot_imm	cvlt_tot_recall	0.99
Music audiation, musicality, musical aptitude: tonal accuracy and total accuracy	AMMA	amma_tonal	amma_total	0.99
Music audiation, musicality, musical aptitude: rhythm accuracy and total accuracy	AMMA	amma_rhythm	amma_total	0.99
Reading skills: regular words and total accuracy	Word and Pseudoword Reading	regular_acc	wordreading	0.97
Arithmetic abilities: subtractions accuracy and total accuracy	RTT	rtt_sub_corr	arith	0.96
Fine motor skills: dominant hand taps and total accuracy	Finger Tapping Test	finger_tapping_dominant	fingertap	0.96
Music audiation, musicality, musical aptitude: rhythm accuracy and tonal accuracy	AMMA	amma_tonal	amma_rhythm	0.96
Reading skills: pseudowords and total accuracy	Word and Pseudoword Reading	pseudo_acc	wordreading	0.95
Fine motor skills: non-dominant hand taps and total accuracy	Finger Tapping Test	finger_tapping_nondominant	fingertap	0.95
Arithmetic abilities: additions accuracy and total accuracy	RTT	rtt_sum_corr	arith	0.95
Fine motor skills: both hands accuracy and accuracy across all single-tool trials	Purdue Pegboard Test	purdue_both_avg	purdue_dh_ndh_both_avg	0.91
Reading skills: irregular words and total accuracy	Word and Pseudoword Reading	irregular_acc	wordreading	0.91
Fine motor skills: non-dominant hand and accuracy across all single-tool trials	Purdue Pegboard Test	purdue_ndh_avg	purdue_dh_ndh_both_avg	0.9
Reading skills: regular and irregular words RT	Word and Pseudoword Reading	regular_rt	irregular_rt	0.9
Reading skills: regular and pseudowords accuracy	Word and Pseudoword Reading	regular_acc	pseudo_acc	0.89
Fine motor skills: dominant hand and accuracy across all single-tool trials	Purdue Pegboard Test	purdue_dh_avg	purdue_dh_ndh_both_avg	0.88
Reading skills: regular and irregular words accuracy	Word and Pseudoword Reading	regular_acc	irregular_acc	0.88
Arithmetic abilities: additions and subtractions RT	RTT	rtt_sub_rt	rtt_sum_rt	0.88
Auditory/verbal working memory: backward span and total span	Digit span	digit_back_span	span_verbal	0.87
Reading skills: regular and pseudowords RT	Word and Pseudoword Reading	regular_rt	pseudo_rt	0.85

Language analytic abilities / Pattern recognition: block 2 and block 3 RT	Brocanto	brocanto_rt2	brocanto_rt3	0.85
Spelling skills: irregular words and total accuracy	Spelling Task	spelling_irregular_acc	spelling_tot_acc	0.83
Fine motor skills: dominant and non-dominant hand taps	Finger Tapping Test	finger_tapping_dominant	finger_tapping_nondominant	0.83
Arithmetic abilities: additions and subtractions accuracy	RTT	rtt_sub_corr	rtt_sum_corr	0.82
Auditory/verbal working memory: forward span and total span	Digit span	digit_for_span	span_verbal	0.82
Fine motor skills: non-dominant hand and both hands accuracy	Purdue Pegboard Test	purdue_ndh_avg	purdue_both_avg	0.78
Visuospatial memory: forward span and total span	Corsi Block	corsi_for_span	span_visual	0.78
Reading skills: pseudowords and irregular RT	Word and Pseudoword Reading	irregular_rt	pseudo_rt	0.77
Fine motor skills: accuracy across all single-tool trials and assembly	Purdue Pegboard Test	purdue_dh_ndh_both_avg	purdue_assembly_avg	0.76
Visuospatial memory: backward span and total span	Corsi Block	corsi_back_span	span_visual	0.76
Fine motor skills: both hands accuracy and assembly accuracy	Purdue Pegboard Test	purdue_both_avg	purdue_assembly_avg	0.75
Reading skills: pseudowords and irregular accuracy	Word and Pseudoword Reading	irregular_acc	pseudo_acc	0.75
Language analytic abilities / Pattern recognition: block 2 and overall accuracy	Brocanto	brocanto_corr2	brocanto	0.74
Language analytic abilities / Pattern recognition: block 3 and overall accuracy	Brocanto	brocanto_corr3	brocanto	0.74
Spelling skills: regular words and total accuracy	Spelling Task	spelling_regular_acc	spelling_tot_acc	0.73
Language analytic abilities / Pattern recognition: block 1 and overall accuracy	Brocanto	brocanto_corr1	brocanto	0.73
Reading skills: test reading and regular word reading RT*	Text reading & Word and Pseudoword Reading	reading_text_rt	regular_rt	0.72
Spelling skills: pseudowords and total accuracy	Spelling Task	spelling_pseudo_acc	spelling_tot_acc	0.72
Language analytic abilities / Pattern recognition: block 1 and block 2 RT	Brocanto	brocanto_rt1	brocanto_rt2	0.72
Phonological working memory: span and accuracy	Non-word repetition	non_word_rep_span	non_word_rep_acc	0.71
Fine motor skills: dominant hand and both hands accuracy	Purdue Pegboard Test	purdue_dh_avg	purdue_both_avg	0.7
Reading skills: test reading and irregular word reading RT*	Text reading & Word and Pseudoword Reading	reading_text_rt	irregular_rt	0.69
Reading skills: test reading and pseudoword reading RT*	Text reading & Word and Pseudoword Reading	reading_text_rt	pseudo_rt	0.68
Fine motor skills: dominant hand and assembly accuracy	Purdue Pegboard Test	purdue_dh_avg	purdue_assembly_avg	0.67

A. Rampinini, I. Balboni, O. Kepinska, R. Berthele, N. Golestani

Naming automatisation and Reading skills: total RT and regular word reading RT*	Rapid Automatised Naming & Word and Pseudoword reading	ran_tot_rt	regular_rt	0.67
Fine motor skills: dominant hand and non-dominant hand accuracy	Purdue Pegboard Test	purdue_dh_avg	purdue_ndh_avg	0.66
Language analytic abilities / Pattern recognition: block 1 and block 3 RT	Brocanto	brocanto_rt1	brocanto_rt3	0.66
Naming automatisation and Reading skills: automatic naming total accuracy and irregular words RT*	Rapid Automatised Naming & Word and Pseudoword reading	ran_tot_acc	irregular_acc	0.64
Language analytic abilities / Pattern recognition: block 2 and block 3 accuracy	Brocanto	brocanto_corr2	brocanto_corr3	0.64
Naming automatisation and Reading skills: automatic naming total RT and irregular words RT*	Rapid Automatised Naming & Word and Pseudoword reading	ran_tot_rt	irregular_rt	0.63
Fine motor skills: non-dominant hand and assembly accuracy	Purdue Pegboard Test	purdue_ndh_avg	purdue_assembly_avg	0.62
Naming automatisation and Reading skills: automatic naming total and irregular words accuracy*	Rapid Automatised Naming & Word and Pseudoword reading	ran_tot_acc	regular_acc	0.62
Motivation and attitude towards foreign languages: ideal L2 self and instrumentality scores	MFQ	ideal_l2_self	instrumentality	0.62
Naming automatisation and Reading skills: automatic naming and word reading total accuracies*	Rapid Automatised Naming & Word and Pseudoword reading	ran_tot_acc	wordreading	0.59
Naming automatisation and Reading skills: automatic naming and text reading total RT*	Rapid Automatised Naming & Text reading	ran_tot_rt	reading_text_rt	0.59
Naming automatisation and Reading skills: automatic naming and pseudowords RT*	Rapid Automatised Naming & Word and Pseudoword reading	ran_tot_rt	pseudo_rt	0.59
Motivation and attitude towards foreign languages: ideal L2 self and international contact scores	MFQ	ideal_l2_self	intl_contact	0.57
Phonological awareness RT *	Spoonerisms & Phoneme suppression	spoon_rt_manual	phon_suppr_rt_manual	0.56
Motivation and attitude towards foreign languages: ideal L2 self and interest in foreign languages scores	MFQ	ideal_l2_self	l2_interest	0.56
Spelling skills: regular and irregular words accuracy	Spelling Task	spelling_regular_acc	spelling_irregular_acc	0.55
Code switching habits: contextual and involuntary switching	Code Switching	swt_score_cs	swt_score_us	0.55
Naming automatisation and Reading skills: automatic naming and nonword repetition accuracy*	Rapid Automatised Naming & Nonword Repetition	ran_tot_acc	non_word_rep_acc	0.52
Language analytic abilities / Pattern recognition block 1 accuracy & Verbal working memory (long term recall)*	Brocanto & CVLT	brocanto_corr1	cvlt_long_corr	0.52
Language analytic abilities / Pattern recognition total accuracy & Verbal working memory (long term recall)*	Brocanto & CVLT	cvlt_long_corr	brocanto	0.51
Music audiation, musicality, musical aptitude and Musical experience: tonal accuracy and musical training*	AMMA & MUSEBAQ	amma_tonal	ind_mus_train_imt	0.5
Music audiation, musicality, musical aptitude and Musical experience: total accuracy and musical training*	AMMA & MUSEBAQ	amma_total	ind_mus_train_imt	0.5

Reading skills: test reading accuracy and irregular word reading RT**	Text reading & Word and Pseudoword Reading	reading_text_acc	irregular_rt	-0.51
Reading skills: test reading RT and Spelling skills total accuracy**	Text reading & Spelling test	reading_text_rt	spelling_tot_acc	-0.51
Reading skills: test reading RT and Spelling skills irregular words accuracy**	Text reading & Spelling test	reading_text_rt	spelling_irregular_acc	-0.52
Rote learning: accuracy and RT	MLAT5	mlat5_corr	mlat5_rt	-0.55
Reading skills: test reading accuracy and regular word reading RT**	Text reading & Word and Pseudoword Reading	reading_text_acc	regular_rt	-0.59
Reading skills: test reading accuracy and pseudoword reading RT**	Text reading & Word and Pseudoword Reading	reading_text_acc	pseudo_rt	-0.61
Motivation and attitude towards foreign languages: anxiety and confidence scores	MFQ	l2_anxiety	l2_confidence	-0.72
Arithmetic abilities: subtractions accuracy and additions RT	RTT	rtt_sub_corr	rtt_sum_rt	-0.78
Arithmetic abilities: subtractions RT and additions accuracy	RTT	rtt_sub_rt	rtt_sum_corr	-0.81
Arithmetic abilities: additions accuracy and RT	RTT	rtt_sum_corr	rtt_sum_rt	-0.86
Arithmetic abilities: additions RT and total accuracy	RTT	rtt_sum_rt	arith	-0.86
Arithmetic abilities: subtractions RT and total accuracy	RTT	rtt_sub_rt	arith	-0.9
Arithmetic abilities: subtractions accuracy and RT	RTT	rtt_sub_corr	rtt_sub_rt	-0.91

Table S3: Internal consistency values measured via Cronbach alpha, where a >= .5. Lower and upper bounds are reported at a 95% confidence interval. For clarity, values where a = > .6 are in bold.

Test	Metric	Cronbach alpha	95% CI lower bound	95% CI upper bound
Finger tapping: non-dominant hand	**finger-tapping_nondominant**	**0.98**	0.97	0.99
Finger tapping: dominant hand	**finger-tapping_dominant**	**0.97**	0.95	0.98
Brocanto: RT block 3	**brocanto_rt_3**	**0.96**	0.94	0.97
Brocanto: RT block 2	**brocanto_rt_2**	**0.96**	0.95	0.97
Brocanto: RT block 1	**brocanto_rt_1**	**0.95**	0.93	0.96
Purdue pegboard: assembly score	**purdue_assembly**	**0.93**	0.90	0.95
Text reading: RT	**reading_rt**	**0.93**	0.89	0.95
Rapid automatised naming: RT	**ran_rt**	**0.93**	0.91	0.95
MUSEBAQ: engaged (music) production score	**engaged_production**	**0.92**	0.89	0.94
MUSEBAQ: dance factor	**dance**	**0.90**	0.85	0.93
CVLT: total recall score	**cvlt_tot-recall**	**0.90**	0.87	0.93
Code switching: contextual	**swt_CS**	**0.89**	0.84	0.92
Purdue pegboard: both hands score	**purdue_both**	**0.89**	0.84	0.92
CVLT: total immediate recall score	**cvlt_tot-imm**	**0.88**	0.84	0.91
Raven's Advanced Progressive Matrices: RT	**apm_rt**	**0.88**	0.85	0.91
RTT: subtractions accuracy	**rtt-sub_corr**	**0.86**	0.82	0.90
MFQ: foreign language usage willingness	**usage_willingness**	**0.85**	0.80	0.89
MLAT5: RT	**mlat5_rt**	**0.85**	0.81	0.89
Purdue pegboard: dominant hand	**purdue_DH**	**0.85**	0.80	0.90
Artgram: RT	**artgram_rt**	**0.84**	0.79	0.88
MUSEBAQ: social connection factor	**social_connection**	**0.84**	0.77	0.89
RTT: additions accuracy	**rtt-sum_corr**	**0.84**	0.80	0.89
Purdue pegboard: non-dominant hand	**purdue_NDH**	**0.83**	0.77	0.88
CVLT: recognition RT	**cvlt_reco_rt**	**0.82**	0.76	0.87
MLAT5: accuracy	**mlat5_corr**	**0.82**	0.77	0.87
MLAT5: errors	**mlat5_incorr**	**0.82**	0.77	0.87
IRQ: visual factor	**irq_visual**	**0.81**	0.75	0.86
Adult Reading History score	**ahrq**	**0.81**	0.75	0.86
CVLT: recognition accuracy	**cvlt_reco_corr**	**0.81**	0.76	0.86
Brocanto accuracy: block 3	**brocanto_corr_3**	**0.80**	0.74	0.85
IRQ: verbal factor	**irq_verbal**	**0.78**	0.71	0.84
MFQ: international contact	**intl_contact**	**0.78**	0.70	0.84
MFQ: confidence using foreign languages	**l2_confidence**	**0.77**	0.69	0.84
MFQ: foreign language environment	**milieu**	**0.76**	0.68	0.83
MUSEBAQ: cognitive and emotional regulation factor	**cognitive_emotional_regulation**	**0.72**	0.63	0.80

Raven's Advanced Progressive Matrices: accuracy	**apm_corr**	**0.72**	0.64	0.79
Text reading: accuracy	**reading_acc**	**0.72**	0.59	0.81
MFQ: self idealisation as foreign language speaker	**ideal_l2_self**	**0.71**	0.61	0.79
Brocanto accuracy: block 2	**brocanto_corr_2**	**0.70**	0.61	0.78
Hindi dental-retroflex task	**hindi**	**0.70**	0.61	0.78
MFQ: anxiety towards foreign languages	**l2_anxiety**	**0.69**	0.57	0.78
Code switching: unwilling/involuntary	**swt_US**	**0.68**	0.56	0.78
MUSEBAQ: physical exercise factor	**physical_exercise**	**0.67**	0.53	0.76
MFQ: interest for foreign languages	**l2_interest**	**0.67**	0.55	0.76
IRQ: orthographic factor	**irq_orto**	**0.66**	0.55	0.75
MUSEBAQ: music listening factor	**ind_mus_listening_iml**	**0.66**	0.50	0.77
AMMA: total score	**amma**	**0.65**	0.54	0.74
MFQ: instrumentality of foreign languages	**instrumentality**	**0.65**	0.54	0.75
IRQ: manipulation factor	**irq_manip**	**0.65**	0.54	0.75
Spelling test: accuracy	**spelling**	**0.62**	0.46	0.73
Digit span: backward score	**digit-back**	**0.61**	0.49	0.71
Digit span: forward score	digit-for	0.58	0.44	0.69
MUSEBAQ: index of musical training	ind_mus_train_imt	0.57	0.40	0.70
Corsi Blocks: forward score	corsi-for	0.55	0.42	0.67
Artgram: accuracy	artgram_corr	0.54	0.39	0.66
ANT-I inhibition gain	**ANT-I inhibition**	**0.67*, CI 95%, 0.55, 0.77**		
ANT-I re-orienting gain	**ANT-I re-orienting**	**0.65*, CI 95% 0.51, 0.76**		
ANT-I orienting gain	**ANT-I orienting**	**0.61*, CI 95% 0.46, 0.73**		
ANT-I alerting gain	ANT-I alerting	0.51*, CI 95% 0.32, 0.66		
Farsi uvular production: native likeness score (accuracy)	**Farsi uvular production**	***Inter-rater reliability:*** $r_{pearson}(95)$**: 0.68** $p < .0001$		

A. Rampinini, I. Balboni, O. Kepinska, R. Berthele, N. Golestani

Table S4
Descriptives of the z-scored Questionnaire data.

	Range	*Skewness*	*Kurtosis*
entropy_competence_speak	6.57	-0.20	1.31
entropy_competence_read	6.53	-0.20	1.43
entropy_competence_compr	6.67	-0.11	1.84
entropy_curr_tot_exp	3.92	-0.07	-0.71
ahrq_score	5.60	0.68	0.69
manipulationfactor	5.39	-0.51	0.35
orthohraphicfactor	4.59	0.29	-0.15
verbalfactor	4.35	-0.32	-0.31
visualfactor	5.48	-0.86	1.16
ideal_l2_self	4.38	-0.84	0.20
instrumentality	4.47	-0.55	0.49
intl_contact	4.52	-1.25	1.42
l2_interest	4.91	-0.89	0.75
l2_anxiety	4.31	0.10	-0.63
l2_confidence	4.21	-0.27	-0.32
milieu	4.72	-1.67	3.39
usage_willingness	4.95	-0.52	0.13
ind_mus_train_imt	3.85	0.46	-0.78
ind_mus_listening_iml	3.91	0.24	-0.52
mus_instr_play_imip	9.74	8.70	79.20
cognitive and emotional regulation	4.38	-0.35	-0.31
social connection	4.48	-0.29	-0.42
engaged production	3.52	1.20	0.18
dance	2.96	0.35	-1.36
physical exercise	4.88	-1.01	1.62
hand_index	4.42	-1.94	3.51
bsmss	3.96	-0.45	-0.58

Table S5
Descriptives of the z-scored Task data.

	Range	*Skewness*	*Kurtosis*
purdue_dh_avg	4.22	-0.16	-0.75
purdue_ndh_avg	4.66	0.05	-0.34
purdue_both_avg	5.39	-0.53	0.38
purdue_dh_ndh_both_avg	4.58	-0.39	-0.53
purdue_assembly_avg	5.25	-0.53	0.28
ran_tot_acc	8.07	-4.71	28.72
ran_tot_rt	6.47	1.99	6.45
reading_text_acc	5.92	-2.09	5.54
reading_text_rt	5.46	1.19	2.03

cvlt_tot_imm	4.91	-0.61	0.42
cvlt_tot_recall	4.98	-0.74	0.70
spelling_regular_acc	4.21	-0.92	0.32
spelling_irregular_acc	4.52	-0.38	-0.48
spelling_pseudo_acc	5.02	-0.41	-0.13
spelling_tot_acc	4.19	-0.62	-0.24
spoon_acc	4.94	-2.61	6.76
spoon_rt_manual	4.91	1.78	3.49
regular_acc	8.80	-7.38	56.13
irregular_acc	10.50	-7.06	58.74
pseudo_acc	7.11	-5.41	32.13
regular_rt	6.59	1.72	5.24
irregular_rt	5.46	1.49	2.69
pseudo_rt	5.55	1.28	2.59
phon_suppr_acc	5.29	-2.21	5.78
wordreading	8.74	-6.85	49.85
alerting	4.33	0.33	-0.52
orienting	5.64	0.00	0.17
reorienting	5.35	0.65	0.48
inhibition	5.10	0.47	0.18
rtt_sub_corr	4.54	-0.17	-0.40
rtt_sub_incorr	5.06	1.40	2.21
rtt_sub_rt	5.50	1.05	1.32
finger_tapping_dominant	5.34	0.42	0.85
finger_tapping_nondominant	5.85	0.63	0.76
digit_for_span	4.09	0.19	-0.56
digit_back_span	4.93	0.54	-0.05
rtt_sum_corr	4.34	-0.30	-0.74
rtt_sum_incorr	5.28	1.43	2.80
rtt_sum_rt	6.13	1.48	3.57
corsi_back_span	3.35	0.10	-0.90
brocanto_corr2	4.70	0.20	-0.54
brocanto_incorr2	4.77	-0.12	-0.46
brocanto_rt2	5.11	-0.54	0.39
brocanto_corr3	4.09	0.29	-0.68
brocanto_incorr3	4.18	-0.25	-0.72
brocanto_rt3	5.23	-0.45	0.25
mlat5_corr	4.01	-0.64	-0.26
mlat5_incorr	4.02	0.61	-0.37
mlat5_rt	5.46	1.12	1.64
artgram_corr	5.14	-0.57	0.06
artgram_incorr	5.29	0.74	0.59
artgram_rt	6.59	1.60	4.12

corsi_for_span	4.02	-0.32	-0.45
amma_tonal	4.46	0.15	-0.44
amma_rhythm	4.84	-0.01	-0.10
amma_total	4.69	0.09	-0.29
hindi_score_weighted	4.06	0.84	-0.41
arith	4.54	-0.19	-0.68
fingertap	5.26	0.43	0.41
span_visual	4.78	-0.04	-0.41
span_verbal	4.92	0.64	-0.09

Supplementary information v2.0 OCT-24: NEBULA101: an open dataset for the study of language aptitude in behaviour, brain structure and function

A. Rampinini, I. Balboni, O. Kepinska, R. Berthele, N. Golestani

Figure S2

Correlation matrix of the z-scored behavioural data with only significant correlations highlighted.

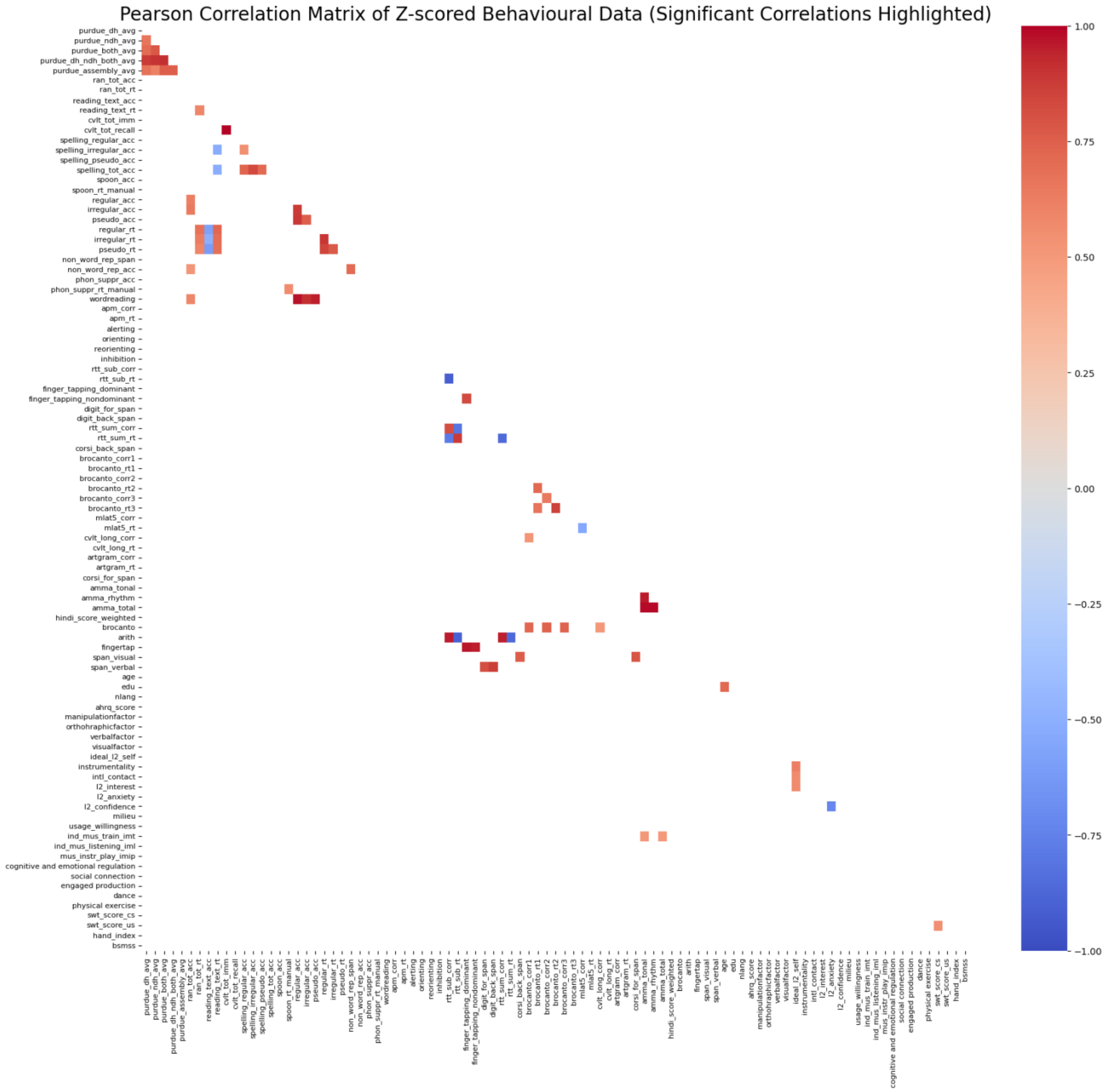

Figure S3

Present and missing data at a glance. Yellow squares indicate present data, purple squares indicate missing data. Subject codes are on the y axis, task names on the x axis.

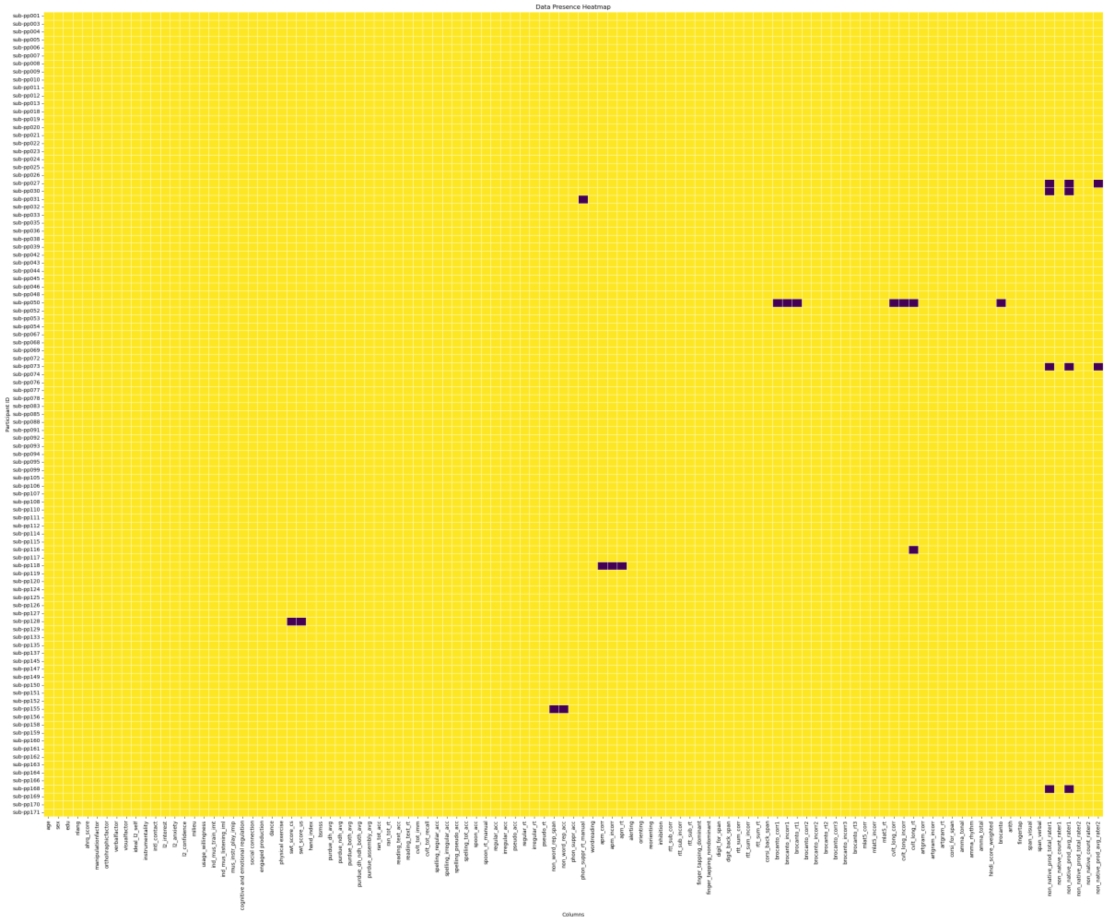

Figure S4. Entropy violin plots: distribution shape of entropy scores.

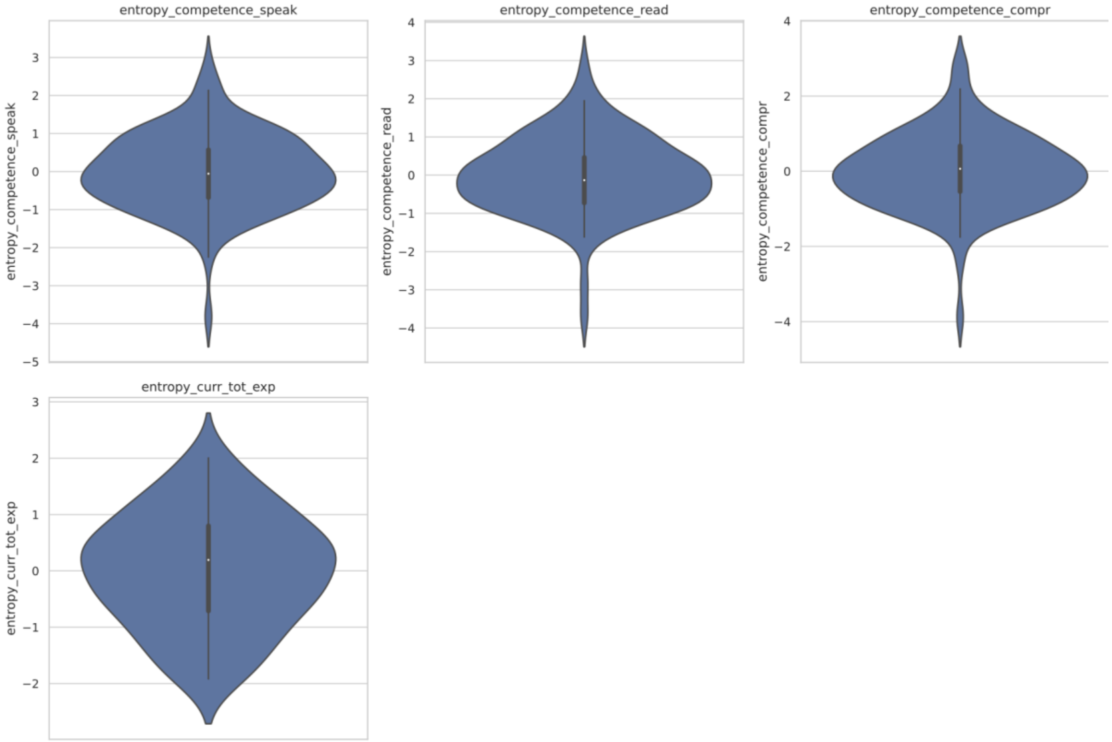

Figure S5. Questionnaire violin plots: distriution shape of questionnaire scores.

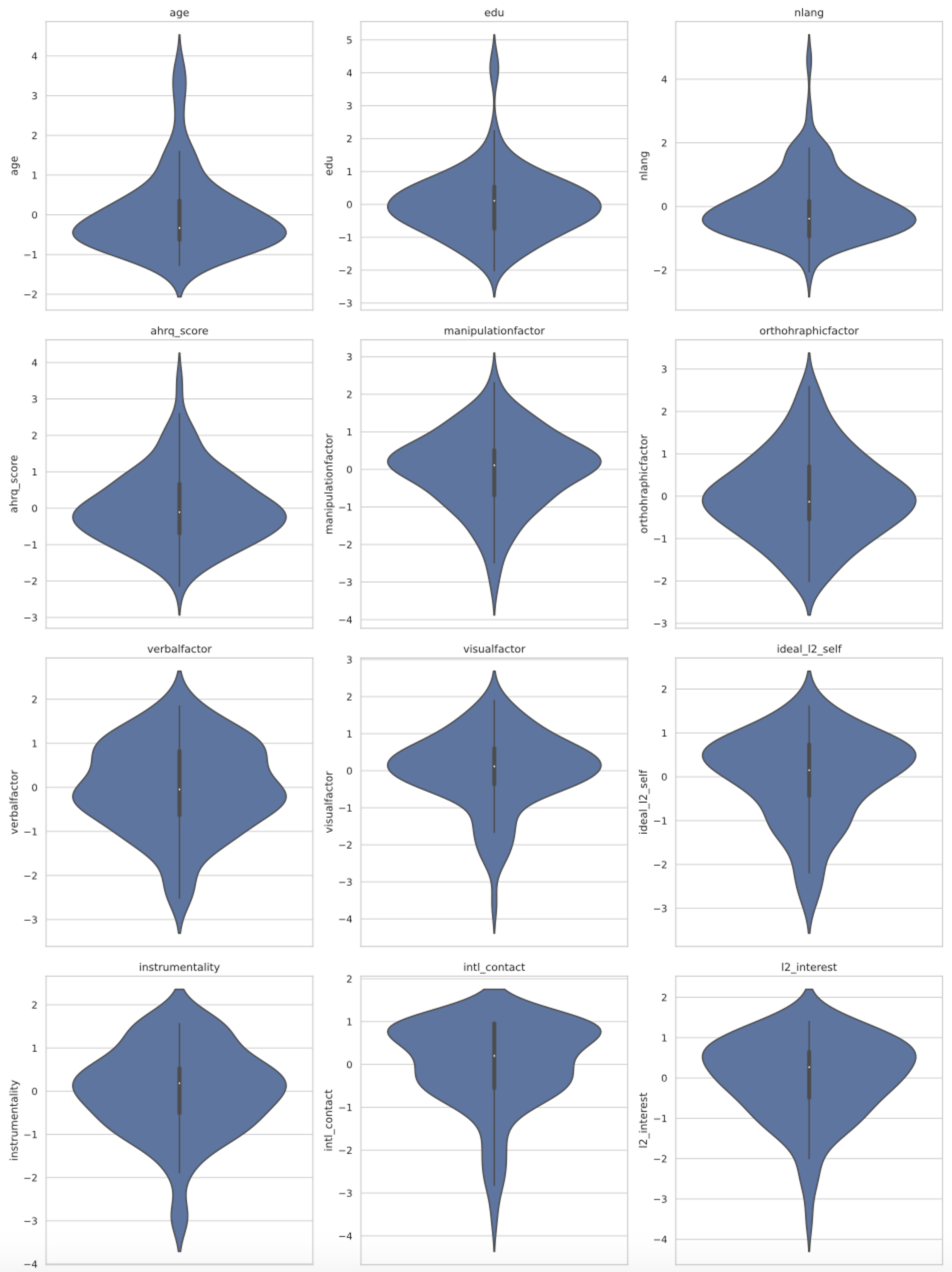

A. Rampinini, I. Balboni, O. Kepinska, R. Berthele, N. Golestani

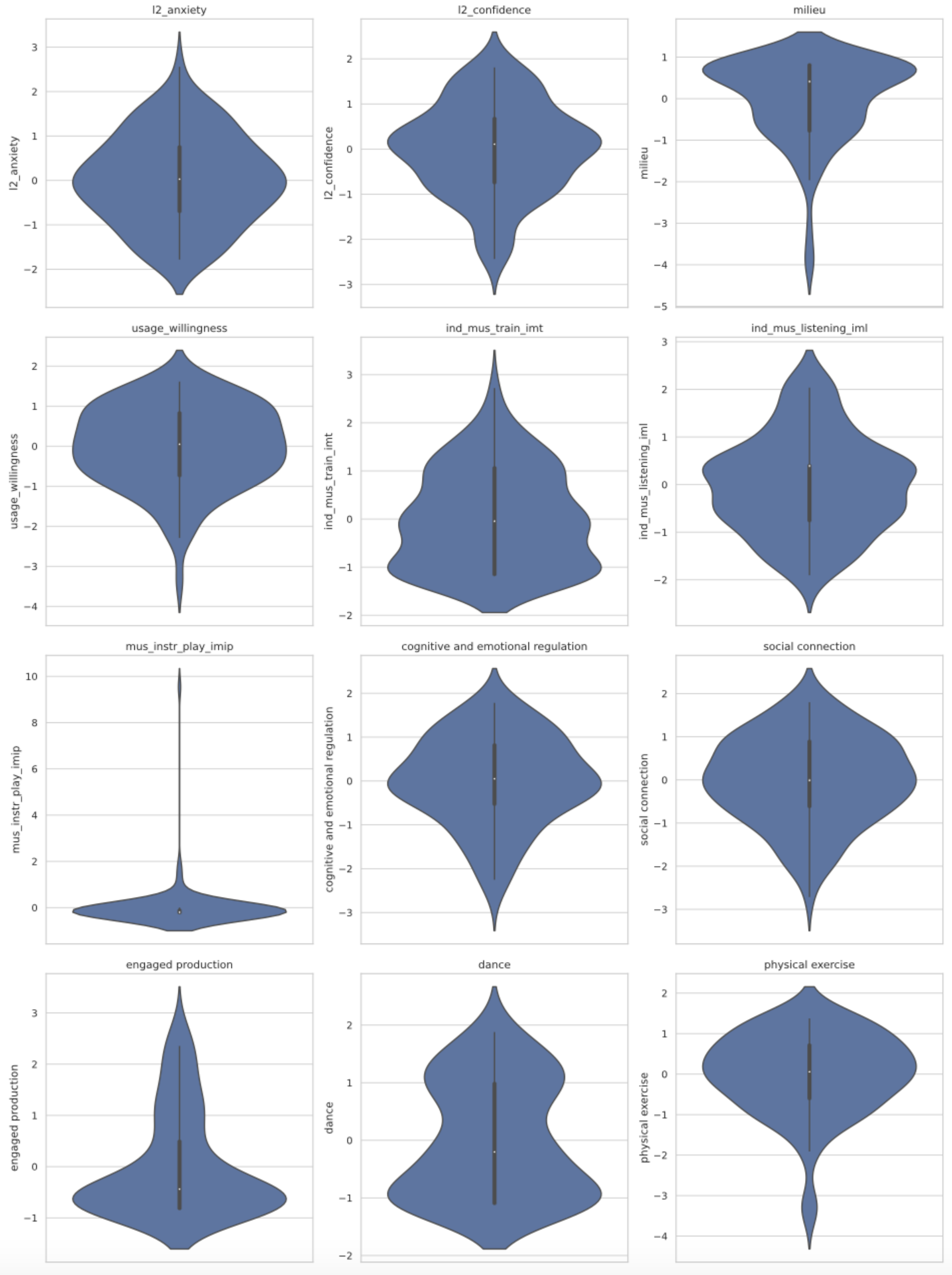

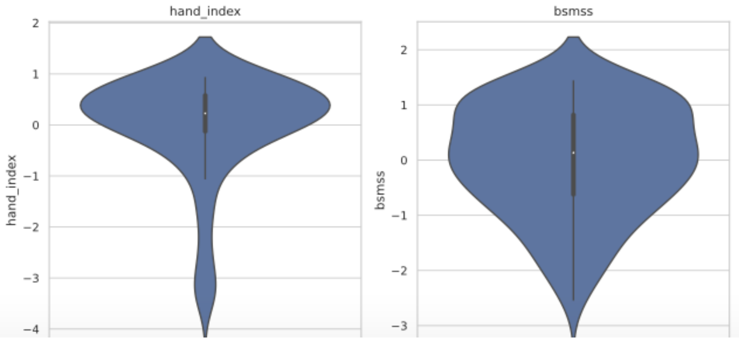
hand_index
2
1
0
−1
−2
−3
−4
hand_index
bsmss
2
1
0
−1
−2
−3
bsmss

Figure S6. Task violin plots. Distribution shape of task scores.

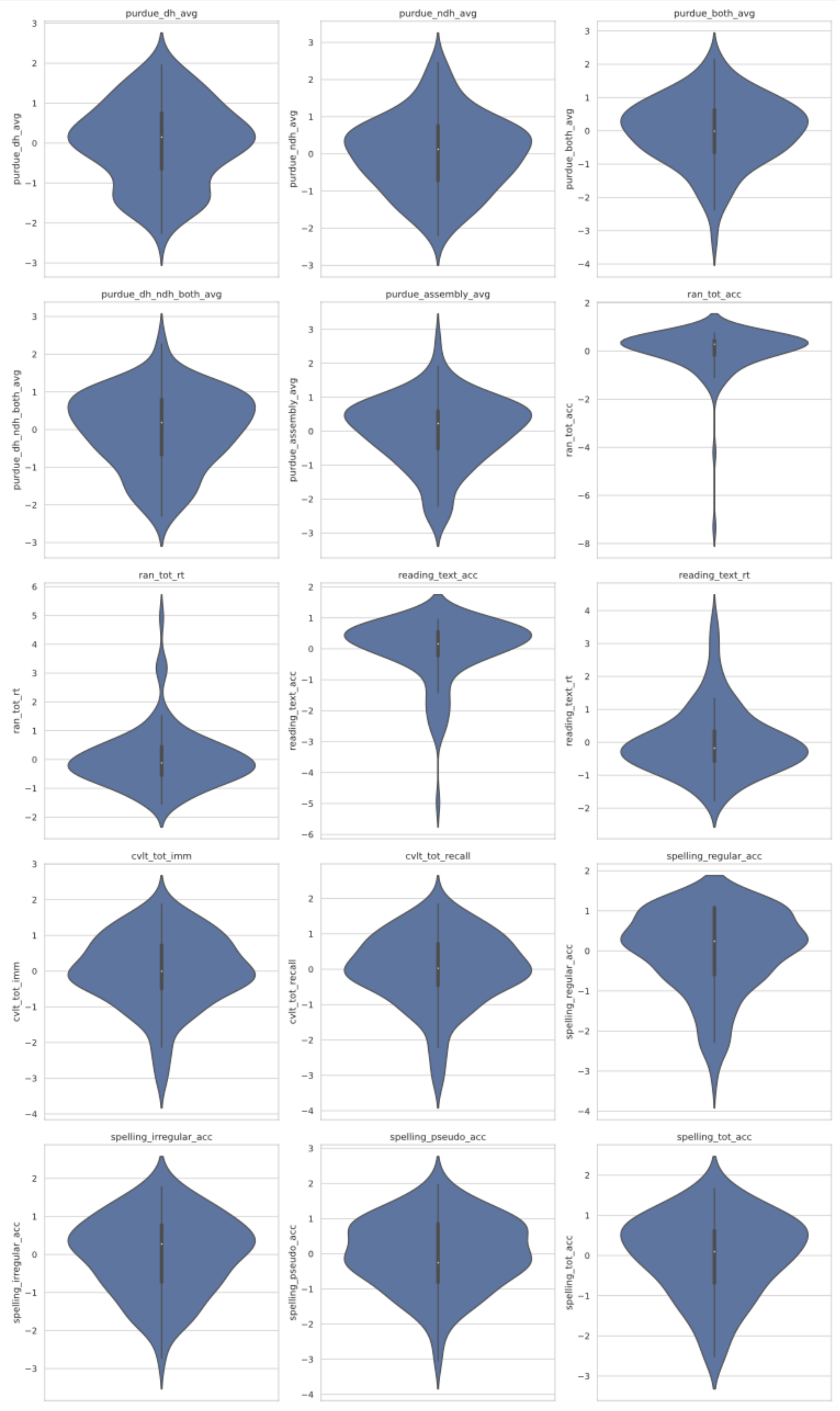

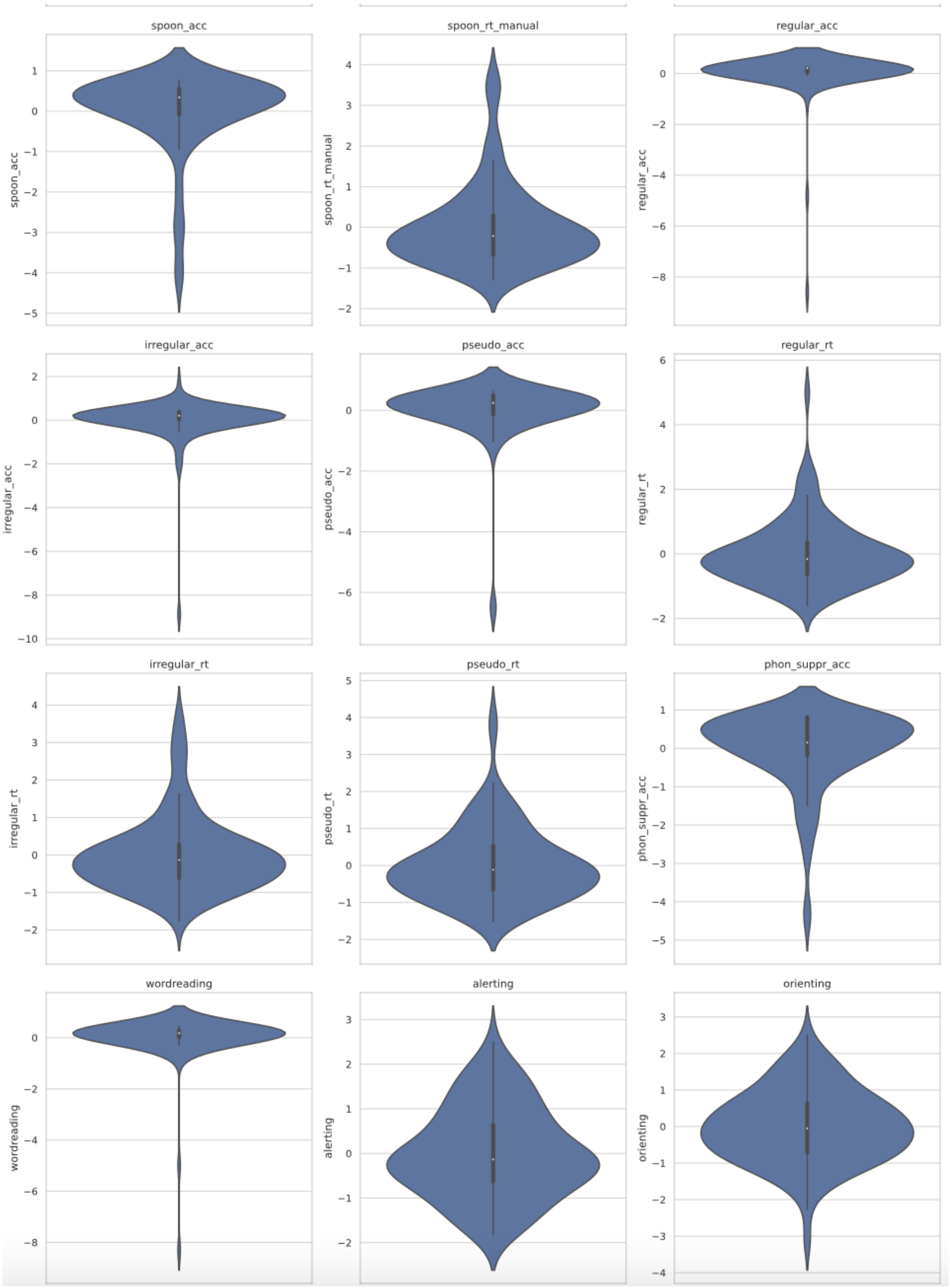
spoon_acc
spoon_rt_manual
regular_acc
irregular_acc
pseudo_acc
regular_rt
irregular_rt
pseudo_rt
phon_suppr_acc
wordreading
alerting
orienting

A. Rampinini, I. Balboni, O. Kepinska, R. Berthele, N. Golestani

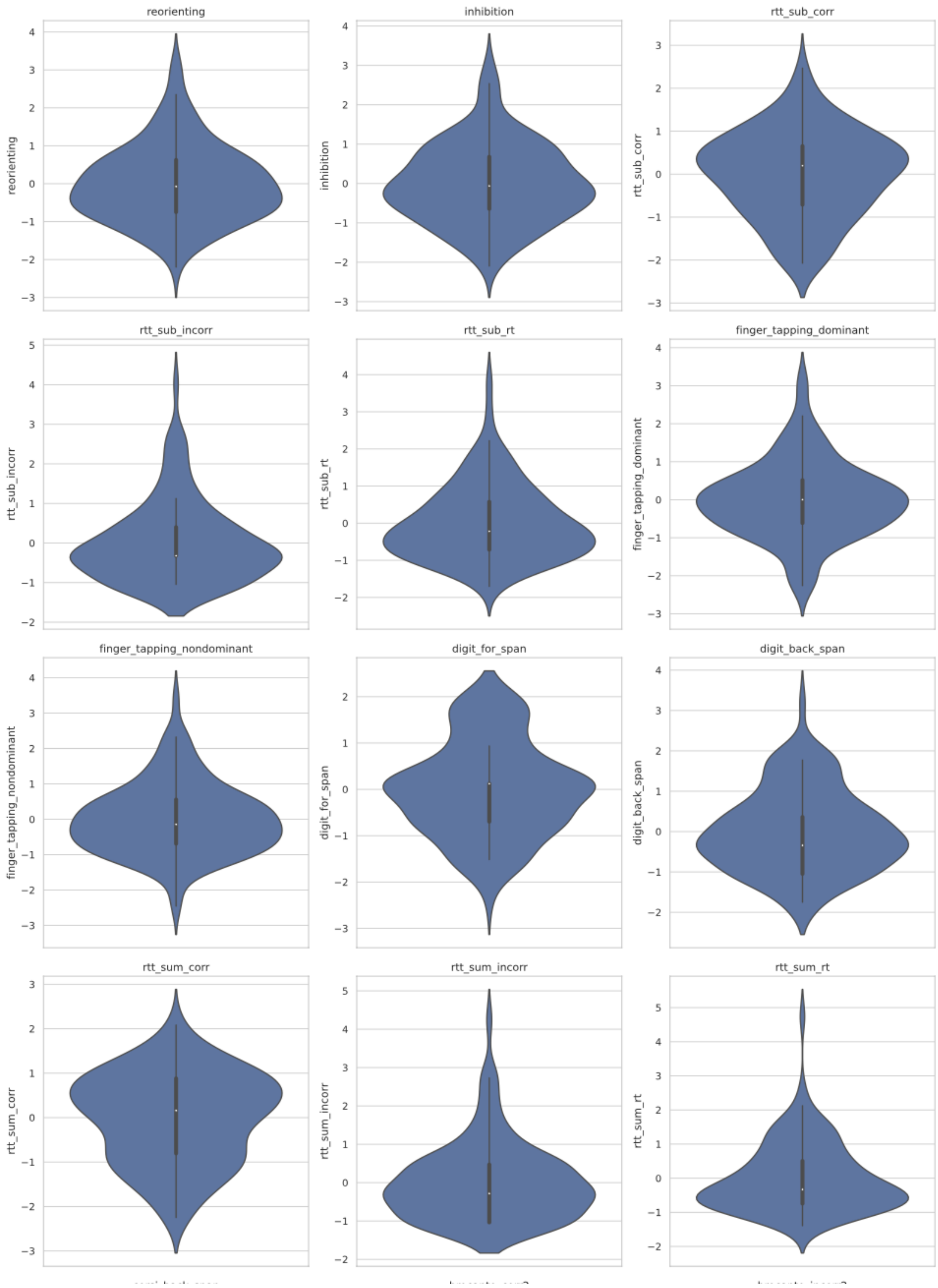

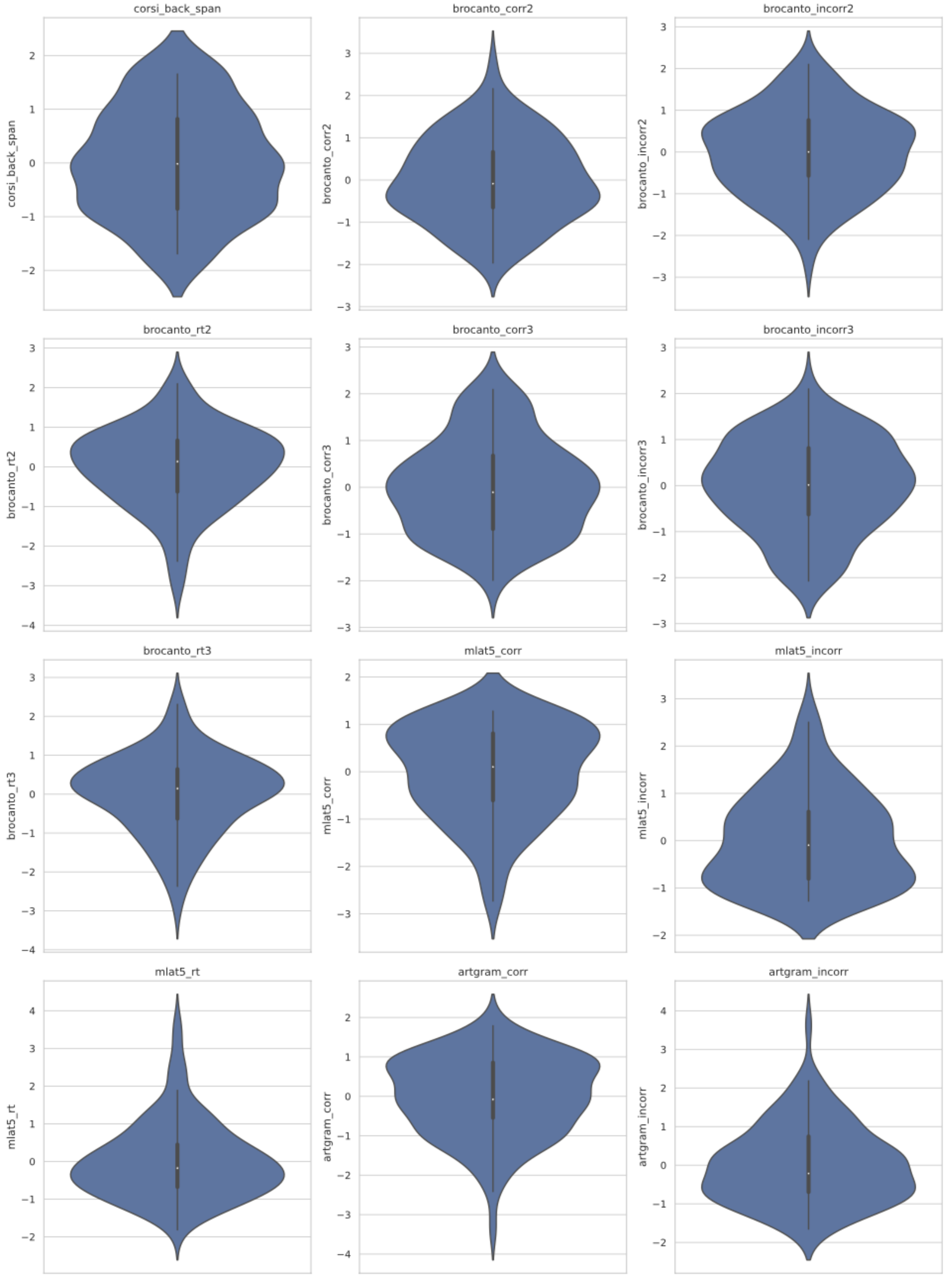
corsi_back_span
corsi_back_span
brocanto_corr2
brocanto_corr2
brocanto_incorr2
brocanto_incorr2
brocanto_rt2
brocanto_rt2
brocanto_corr3
brocanto_corr3
brocanto_incorr3
brocanto_incorr3
brocanto_rt3
brocanto_rt3
mlat5_corr
mlat5_corr
mlat5_incorr
mlat5_incorr
mlat5_rt
mlat5_rt
artgram_corr
artgram_corr
artgram_incorr
artgram_incorr

A. Rampinini, I. Balboni, O. Kepinska, R. Berthele, N. Golestani

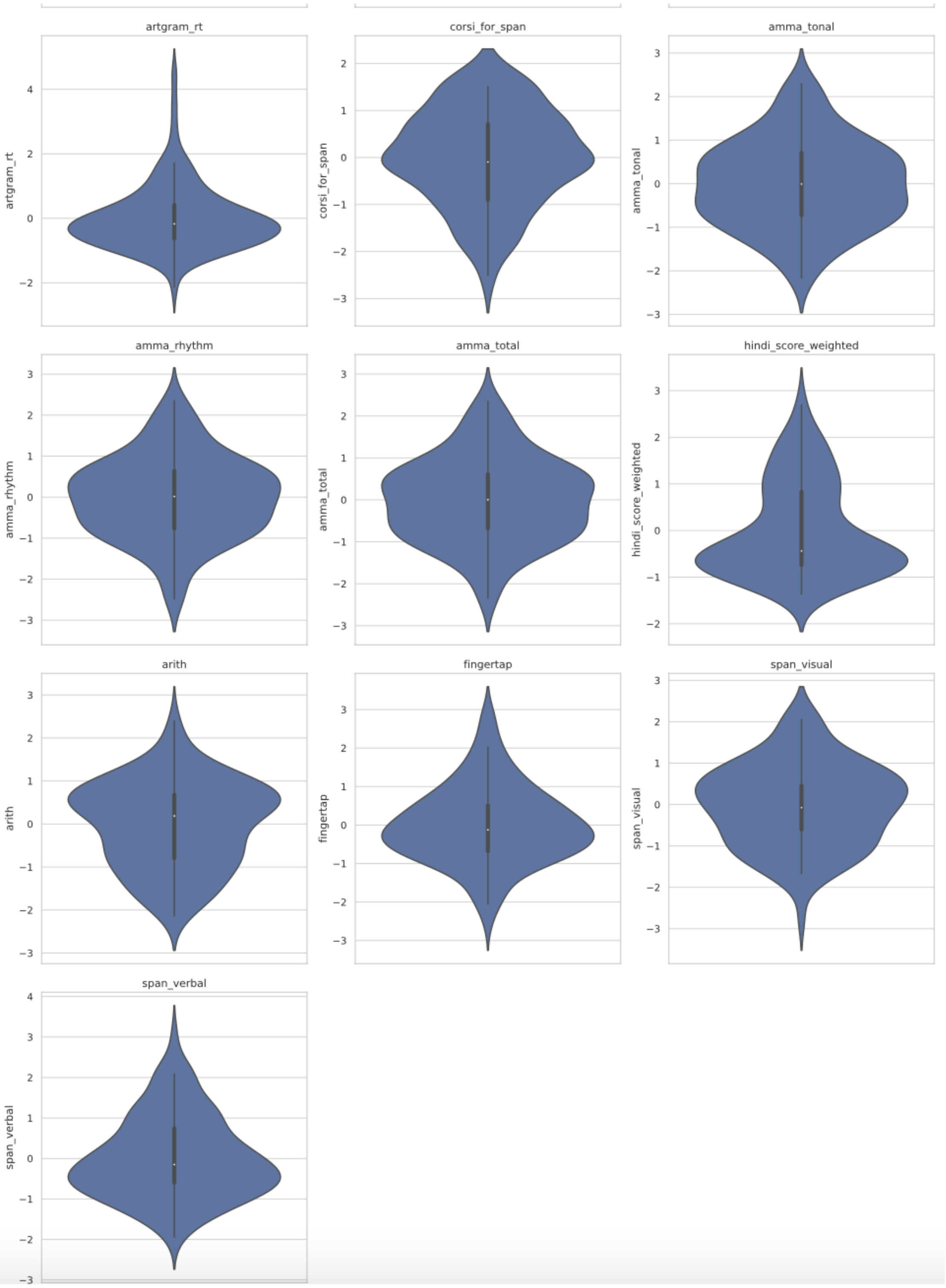

ANTi_reliability_NEBULA101

IB

5/8/2024

Reliability assessment for the task ANT-i was computed with split-half correlations using the package splithalf

Cleaning and preprocessing

We clean and preprocess the data as we did for the scoring but this time create 1 variable containing all of the participants with cleaned scores divided by blocks to be used for the split-half correlation. Max responses per participant 432. Responses missing could be due to lack of attempt, abnormal timing, and incorrect responses are removed

select only relevant pp

```
pp_toinclude=read.delim('/data/team/Aptitude/nebula101/participants.tsv')
final_data <- final_data %>%
  filter(participant_id %in% pp_toinclude$participant_id)
```

##Split-half correlation

```
difference <- splithalf(data = final_data,
                        outcome = "RT",
                        score = "difference",
                        halftype = "random",
                        permutations = 10000,
                        var.RT = "ant_RT",
                        var.participant = "participant_id",
                        var.compare = "alerting.code",
                        compare1 = "0",
                        compare2 = "1",
                        average = "mean",
                        plot = TRUE)
```

```
|
|=================== | 28%
|
|==================== | 29%
|
|===================== | 30%
|
|===================== | 31%
|
|====================== | 32%
|
|======================= | 33%
|
|======================== | 34%
|
|======================== | 35%
|
|========================= | 36%
|
|========================== | 37%
|
|========================== | 38%
|
|=========================== | 39%
|
|============================ | 40%
|
|============================ | 41%
|
|============================= | 42%
|
|============================== | 43%
|
|============================== | 44%
|
|=============================== | 45%
|
|================================ | 46%
|
|================================= | 47%
|
|================================= | 48%
|
|================================== | 49%
|
|=================================== | 50%
|
|==================================== | 51%
|
|===================================== | 52%
|
|===================================== | 53%
|
|====================================== | 54%
|
|======================================= | 55%
```

```
|
|========================================                              |  56%
|
|========================================                              |  57%
|
|=========================================                             |  58%
|
|==========================================                            |  59%
|
|==========================================                            |  60%
|
|===========================================                           |  61%
|
|============================================                          |  62%
|
|============================================                          |  63%
|
|=============================================                         |  64%
|
|==============================================                        |  65%
|
|==============================================                        |  66%
|
|===============================================                       |  67%
|
|================================================                      |  68%
|
|=================================================                     |  69%
|
|=================================================                     |  70%
|
|==================================================                    |  71%
|
|===================================================                   |  72%
|
|===================================================                   |  73%
|
|====================================================                  |  74%
|
|=====================================================                 |  75%
|
|=====================================================                 |  76%
|
|======================================================                |  77%
|
|=======================================================               |  78%
|
|=======================================================               |  79%
|
|========================================================              |  80%
|
|=========================================================             |  81%
|
|==========================================================            |  82%
|
|==========================================================            |  83%
```

```
  |
  |===========================================================           |  84%
  |
  |============================================================          |  85%
  |
  |============================================================          |  86%
  |
  |=============================================================         |  87%
  |
  |==============================================================        |  88%
  |
  |==============================================================        |  89%
  |
  |===============================================================       |  90%
  |
  |================================================================      |  91%
  |
  |================================================================      |  92%
  |
  |=================================================================     |  93%
  |
  |==================================================================    |  94%
  |
  |===================================================================   |  95%
  |
  |===================================================================   |  96%
  |
  |====================================================================  |  97%
  |
  |===================================================================== |  98%
  |
  |===================================================================== |  99%
  |
  |======================================================================| 100%[1] "c
ondition all complete"
## [1] "Calculating split half estimates"
## [1] "split half estimates for 10000 random splits"
##   condition   n spearmanbrown SB_low SB_high
## 1       all 101          0.51   0.32    0.66
## [1] "this could be reported as: using 10000 random splits, the spearman-brown corr
ected reliability  estimate for the all condition was 0.51, 95% CI [0.32, 0.66]"
```

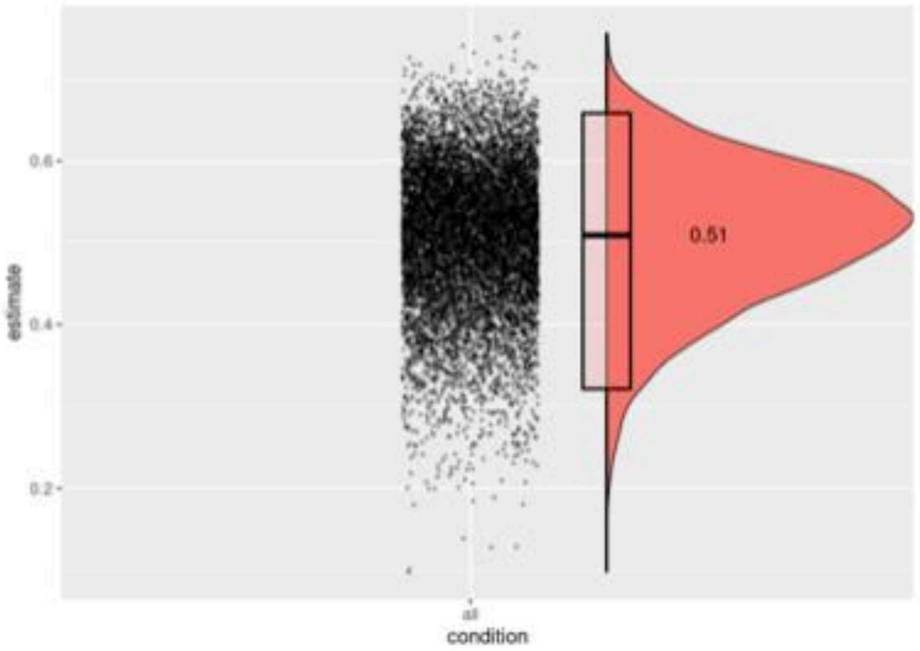


```
difference_orienting <- splithalf(data = final_data,
                        outcome = "RT",
                        score = "difference",
                        halftype = "random",
                        permutations = 10000,
                        var.RT = "ant_RT",
                        var.participant = "participant_id",
                        var.compare = "cued.code",
                        compare1 = "0",
                        compare2 = "1",
                        average = "mean",
                        plot = TRUE)
```

```
##
  |
  |                                                                      |   0%
  |
  |=                                                                     |   2%
  |
  |==                                                                    |   3%
  |
  |===                                                                   |   4%
  |
  |===                                                                   |   5%
  |
  |====                                                                  |   6%
  |
  |=====                                                                 |   7%
  |
  |======                                                                |   8%
  |
  |======                                                                |   9%
  |
  |=======                                                               |  10%
  |
  |========                                                              |  11%
  |
  |========                                                              |  12%
  |
  |=========                                                             |  13%
  |
  |==========                                                            |  14%
  |
  |==========                                                            |  15%
  |
  |===========                                                           |  16%
  |
  |============                                                          |  17%
  |
  |============                                                          |  18%
  |
  |=============                                                         |  19%
  |
  |==============                                                        |  20%
  |
  |===============                                                       |  21%
  |
  |===============                                                       |  22%
  |
  |================                                                      |  23%
  |
  |=================                                                     |  24%
  |
  |=================                                                     |  25%
  |
  |==================                                                    |  26%
  |
  |===================                                                   |  27%
```

```
|
|===================                                                   |  28%
|
|====================                                                  |  29%
|
|=====================                                                 |  30%
|
|=====================                                                 |  31%
|
|======================                                                |  32%
|
|=======================                                               |  33%
|
|========================                                              |  34%
|
|========================                                              |  35%
|
|=========================                                             |  36%
|
|==========================                                            |  37%
|
|==========================                                            |  38%
|
|===========================                                           |  39%
|
|============================                                          |  40%
|
|============================                                          |  41%
|
|=============================                                         |  42%
|
|==============================                                        |  43%
|
|==============================                                        |  44%
|
|===============================                                       |  45%
|
|================================                                      |  46%
|
|=================================                                     |  47%
|
|=================================                                     |  48%
|
|==================================                                    |  49%
|
|===================================                                   |  50%
|
|====================================                                  |  51%
|
|=====================================                                 |  52%
|
|=====================================                                 |  53%
|
|======================================                                |  54%
|
|=======================================                               |  55%
```

```
|
|========================================                              |  56%
|
|========================================                              |  57%
|
|=========================================                             |  58%
|
|==========================================                            |  59%
|
|==========================================                            |  60%
|
|===========================================                           |  61%
|
|============================================                          |  62%
|
|============================================                          |  63%
|
|=============================================                         |  64%
|
|==============================================                        |  65%
|
|==============================================                        |  66%
|
|===============================================                       |  67%
|
|================================================                      |  68%
|
|=================================================                     |  69%
|
|=================================================                     |  70%
|
|==================================================                    |  71%
|
|===================================================                   |  72%
|
|===================================================                   |  73%
|
|====================================================                  |  74%
|
|=====================================================                 |  75%
|
|=====================================================                 |  76%
|
|======================================================                |  77%
|
|=======================================================               |  78%
|
|=======================================================               |  79%
|
|========================================================              |  80%
|
|=========================================================             |  81%
|
|==========================================================            |  82%
|
|==========================================================            |  83%
```

```
  |
  |===========================================================           |  84%
  |
  |============================================================          |  85%
  |
  |============================================================          |  86%
  |
  |=============================================================         |  87%
  |
  |==============================================================        |  88%
  |
  |==============================================================        |  89%
  |
  |===============================================================       |  90%
  |
  |================================================================      |  91%
  |
  |================================================================      |  92%
  |
  |=================================================================     |  93%
  |
  |==================================================================    |  94%
  |
  |===================================================================   |  95%
  |
  |===================================================================   |  96%
  |
  |====================================================================  |  97%
  |
  |===================================================================== |  98%
  |
  |===================================================================== |  99%
  |
  |======================================================================| 100%[1] "c
ondition all complete"
## [1] "Calculating split half estimates"
## [1] "split half estimates for 10000 random splits"
##   condition   n spearmanbrown SB_low SB_high
## 1       all 101          0.61   0.46    0.73
## [1] "this could be reported as: using 10000 random splits, the spearman-brown corr
ected reliability  estimate for the all condition was 0.61, 95% CI [0.46, 0.73]"
```

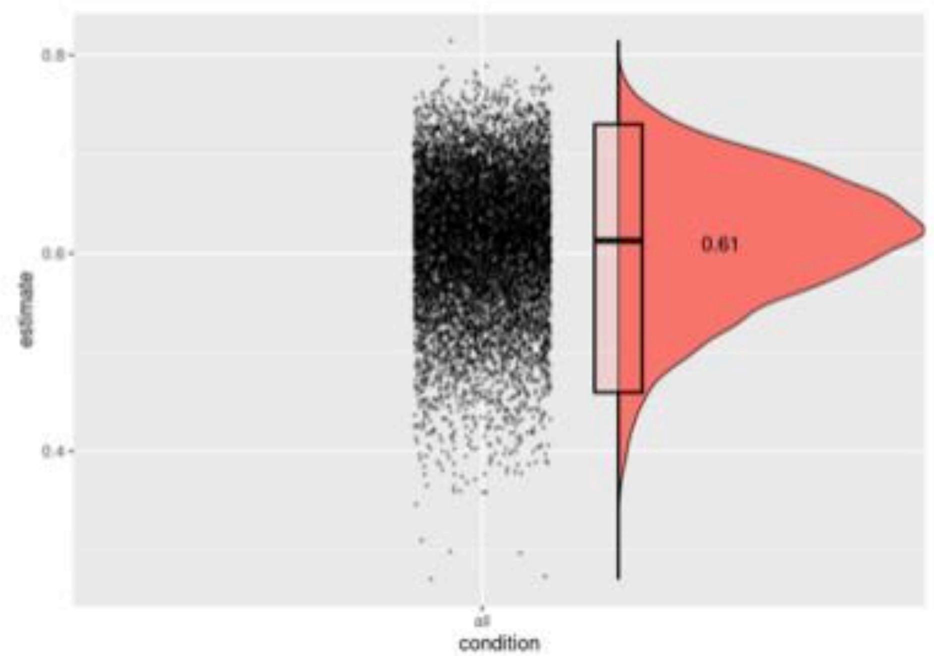


```
difference_reorienting <- splithalf(data = final_data,
                          outcome = "RT",
                          score = "difference",
                          halftype = "random",
                          permutations = 10000,
                          var.RT = "ant_RT",
                          var.participant = "participant_id",
                          var.compare = "cued.code",
                          compare1 = "2",
                          compare2 = "1",
                          average = "mean",
                          plot = TRUE)
```

```
##
  |
  |                                                                      |   0%
  |
  |=                                                                     |   2%
  |
  |==                                                                    |   3%
  |
  |===                                                                   |   4%
  |
  |===                                                                   |   5%
  |
  |====                                                                  |   6%
  |
  |=====                                                                 |   7%
  |
  |======                                                                |   8%
  |
  |======                                                                |   9%
  |
  |=======                                                               |  10%
  |
  |========                                                              |  11%
  |
  |========                                                              |  12%
  |
  |=========                                                             |  13%
  |
  |==========                                                            |  14%
  |
  |==========                                                            |  15%
  |
  |===========                                                           |  16%
  |
  |============                                                          |  17%
  |
  |============                                                          |  18%
  |
  |=============                                                         |  19%
  |
  |==============                                                        |  20%
  |
  |===============                                                       |  21%
  |
  |===============                                                       |  22%
  |
  |================                                                      |  23%
  |
  |=================                                                     |  24%
  |
  |=================                                                     |  25%
  |
  |==================                                                    |  26%
  |
  |===================                                                   |  27%
```

```
|
|===================                                                   |  28%
|
|====================                                                  |  29%
|
|=====================                                                 |  30%
|
|=====================                                                 |  31%
|
|======================                                                |  32%
|
|=======================                                               |  33%
|
|========================                                              |  34%
|
|========================                                              |  35%
|
|=========================                                             |  36%
|
|==========================                                            |  37%
|
|==========================                                            |  38%
|
|===========================                                           |  39%
|
|============================                                          |  40%
|
|============================                                          |  41%
|
|=============================                                         |  42%
|
|==============================                                        |  43%
|
|==============================                                        |  44%
|
|===============================                                       |  45%
|
|================================                                      |  46%
|
|=================================                                     |  47%
|
|=================================                                     |  48%
|
|==================================                                    |  49%
|
|===================================                                   |  50%
|
|====================================                                  |  51%
|
|=====================================                                 |  52%
|
|=====================================                                 |  53%
|
|======================================                                |  54%
|
|=======================================                               |  55%
```

```
  |
  |========================================                              |  56%
  |
  |========================================                              |  57%
  |
  |=========================================                             |  58%
  |
  |==========================================                            |  59%
  |
  |==========================================                            |  60%
  |
  |===========================================                           |  61%
  |
  |============================================                          |  62%
  |
  |============================================                          |  63%
  |
  |=============================================                         |  64%
  |
  |==============================================                        |  65%
  |
  |==============================================                        |  66%
  |
  |===============================================                       |  67%
  |
  |================================================                      |  68%
  |
  |=================================================                     |  69%
  |
  |=================================================                     |  70%
  |
  |==================================================                    |  71%
  |
  |===================================================                   |  72%
  |
  |===================================================                   |  73%
  |
  |====================================================                  |  74%
  |
  |=====================================================                 |  75%
  |
  |=====================================================                 |  76%
  |
  |======================================================                |  77%
  |
  |=======================================================               |  78%
  |
  |=======================================================               |  79%
  |
  |========================================================              |  80%
  |
  |=========================================================             |  81%
  |
  |==========================================================            |  82%
  |
  |==========================================================            |  83%
```

```
  |
  |=======================================================           |  84%
  |
  |========================================================          |  85%
  |
  |========================================================          |  86%
  |
  |=========================================================         |  87%
  |
  |==========================================================        |  88%
  |
  |==========================================================        |  89%
  |
  |===========================================================       |  90%
  |
  |============================================================      |  91%
  |
  |============================================================      |  92%
  |
  |=============================================================     |  93%
  |
  |==============================================================    |  94%
  |
  |==============================================================    |  95%
  |
  |===============================================================   |  96%
  |
  |================================================================  |  97%
  |
  |================================================================  |  98%
  |
  |================================================================= |  99%
  |
  |==================================================================| 100%[1] "c
ondition all complete"
## [1] "Calculating split half estimates"
## [1] "split half estimates for 10000 random splits"
##   condition   n spearmanbrown SB_low SB_high
## 1       all 101          0.65   0.51    0.76
## [1] "this could be reported as: using 10000 random splits, the spearman-brown corr
ected reliability  estimate for the all condition was 0.65, 95% CI [0.51, 0.76]"
```

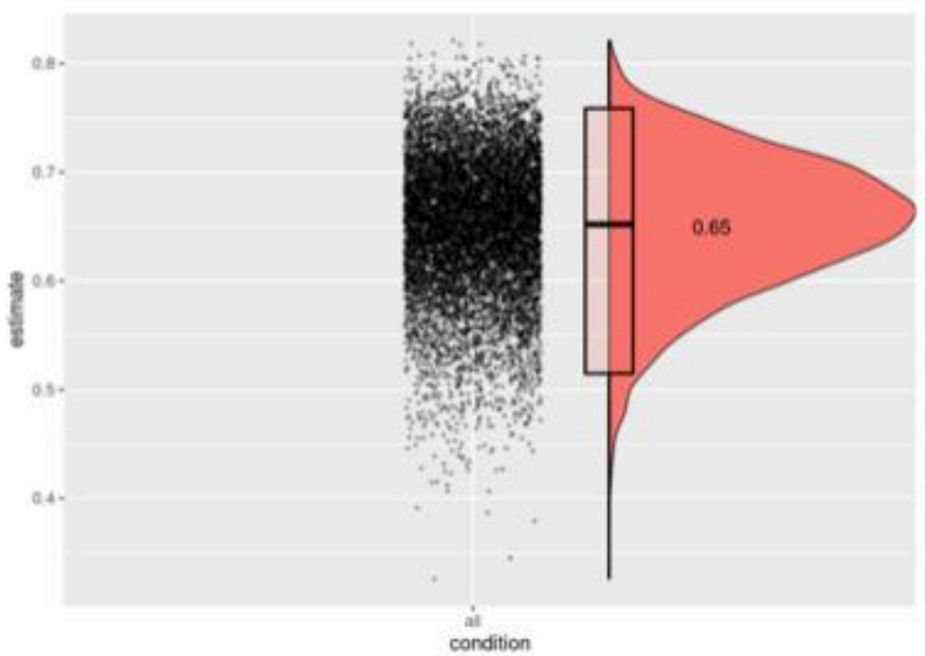


```
difference_executive <- splithalf(data = final_data,
                         outcome = "RT",
                         score = "difference",
                         halftype = "random",
                         permutations = 10000,
                         var.RT = "ant_RT",
                         var.participant = "participant_id",
                         var.compare = "congruency.code",
                         compare1 = "0",
                         compare2 = "1",
                         average = "mean",
                         plot = TRUE)
```

```
##
  |
  |                                                                      |   0%
  |
  |=                                                                     |   2%
  |
  |==                                                                    |   3%
  |
  |===                                                                   |   4%
  |
  |===                                                                   |   5%
  |
  |====                                                                  |   6%
  |
  |=====                                                                 |   7%
  |
  |======                                                                |   8%
  |
  |======                                                                |   9%
  |
  |=======                                                               |  10%
  |
  |========                                                              |  11%
  |
  |========                                                              |  12%
  |
  |=========                                                             |  13%
  |
  |==========                                                            |  14%
  |
  |==========                                                            |  15%
  |
  |===========                                                           |  16%
  |
  |============                                                          |  17%
  |
  |============                                                          |  18%
  |
  |=============                                                         |  19%
  |
  |==============                                                        |  20%
  |
  |===============                                                       |  21%
  |
  |===============                                                       |  22%
  |
  |================                                                      |  23%
  |
  |=================                                                     |  24%
  |
  |=================                                                     |  25%
  |
  |==================                                                    |  26%
  |
  |===================                                                   |  27%
```

```
|
|===================                                                   |  28%
|
|====================                                                  |  29%
|
|=====================                                                 |  30%
|
|=====================                                                 |  31%
|
|======================                                                |  32%
|
|=======================                                               |  33%
|
|========================                                              |  34%
|
|========================                                              |  35%
|
|=========================                                             |  36%
|
|==========================                                            |  37%
|
|==========================                                            |  38%
|
|===========================                                           |  39%
|
|============================                                          |  40%
|
|============================                                          |  41%
|
|=============================                                         |  42%
|
|==============================                                        |  43%
|
|==============================                                        |  44%
|
|===============================                                       |  45%
|
|================================                                      |  46%
|
|=================================                                     |  47%
|
|=================================                                     |  48%
|
|==================================                                    |  49%
|
|===================================                                   |  50%
|
|====================================                                  |  51%
|
|=====================================                                 |  52%
|
|=====================================                                 |  53%
|
|======================================                                |  54%
|
|=======================================                               |  55%
```

```
|
|========================================                              |  56%
|
|========================================                              |  57%
|
|=========================================                             |  58%
|
|==========================================                            |  59%
|
|==========================================                            |  60%
|
|===========================================                           |  61%
|
|============================================                          |  62%
|
|============================================                          |  63%
|
|=============================================                         |  64%
|
|==============================================                        |  65%
|
|==============================================                        |  66%
|
|===============================================                       |  67%
|
|================================================                      |  68%
|
|=================================================                     |  69%
|
|=================================================                     |  70%
|
|==================================================                    |  71%
|
|===================================================                   |  72%
|
|===================================================                   |  73%
|
|====================================================                  |  74%
|
|=====================================================                 |  75%
|
|=====================================================                 |  76%
|
|======================================================                |  77%
|
|=======================================================               |  78%
|
|=======================================================               |  79%
|
|========================================================              |  80%
|
|=========================================================             |  81%
|
|==========================================================            |  82%
|
|==========================================================            |  83%
```

```
  |
  |=======================================================           |  84%
  |
  |========================================================          |  85%
  |
  |========================================================          |  86%
  |
  |=========================================================         |  87%
  |
  |==========================================================        |  88%
  |
  |==========================================================        |  89%
  |
  |===========================================================       |  90%
  |
  |============================================================      |  91%
  |
  |============================================================      |  92%
  |
  |=============================================================     |  93%
  |
  |==============================================================    |  94%
  |
  |===============================================================   |  95%
  |
  |===============================================================   |  96%
  |
  |================================================================  |  97%
  |
  |================================================================= |  98%
  |
  |================================================================= |  99%
  |
  |==================================================================| 100%[1] "c
ondition all complete"
## [1] "Calculating split half estimates"
## [1] "split half estimates for 10000 random splits"
##   condition   n spearmanbrown SB_low SB_high
## 1       all 101          0.67   0.55    0.77
## [1] "this could be reported as: using 10000 random splits, the spearman-brown corr
ected reliability  estimate for the all condition was 0.67, 95% CI [0.55, 0.77]"
```

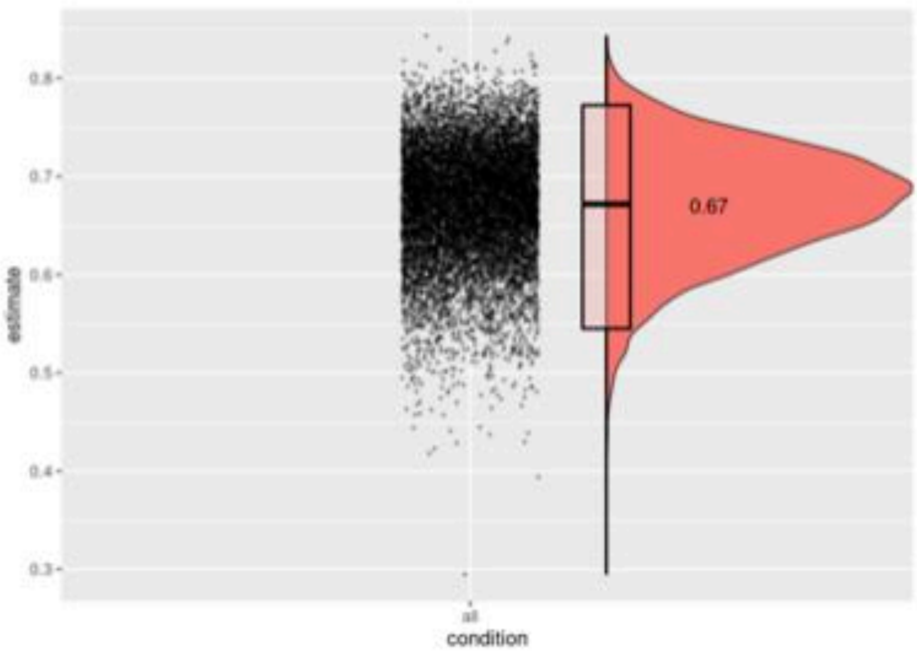
0.8
0.7
0.6
0.5
0.4
0.3
estimate
0.67
all
condition
